# The Validity of the Single-Leg Heel Raise Test in People With Multiple Sclerosis: A Cross-Sectional Study

**DOI:** 10.3389/fneur.2021.650297

**Published:** 2021-07-21

**Authors:** Mark M. Mañago, Paul W. Kline, Michael O. Harris-Love, Cory L. Christiansen

**Affiliations:** ^1^Physical Therapy Program, Department of Physical Medicine and Rehabilitation, School of Medicine, University of Colorado Anschutz Medical, Aurora, CO, United States; ^2^Department of Neurology, School of Medicine, University of Colorado Anschutz Medical, Aurora, CO, United States; ^3^Geriatric, Research, Education, and Clinical Center, VA Eastern Colorado Healthcare System, Aurora, CO, United States; ^4^Department of Physical Therapy, High Point University, One University Parkway, High Point, NC, United States

**Keywords:** multiple sclerosis, rehabilitation, strength, muscle performance, functional mobility

## Abstract

**Background:** The single-leg heel raise test is a common clinical assessment; however, little is known about its validity in people with multiple sclerosis (MS). This study investigated the validity of the single-leg heel raise test in a group of people with MS and a healthy control group (CTL).

**Materials and Methods:** Twenty-one people with MS (49 ± 12 years, Expanded Disability Status Scale 1.5–5.5) and 10 healthy controls (48 ± 12 years) performed the single-leg heel raise test, ankle plantarflexion isometric strength assessment using electromechanical dynamometry, and mobility measures (Timed 25-Foot Walk, 2-Min Walk Test, Functional Stair Test).

**Results:** Convergent validity between the heel raise test and strength was moderate for participants with MS completing <20 heel raises (*r* = 0.63, *p* = 0.001) but weak for the entire sample (*r* = 0.30, *p* = 0.020). Compared to the average CTL group values, the heel raise test differentiated between groups on the MS groups' weaker (*p* < 0.001) and stronger (*p* = 0.003) limbs, while strength only differentiated between groups on the weaker limb (*p* = 0.010). Considering the weaker and strong limbs from the MS group and the CTL group average values, the mobility measures had moderate-to-strong correlations with the heel raise test on the weaker MS limb + CTL (*r* = 0.71–0.78) and stronger MS limb + CTL (*r* = 0.62–0.70), and weak-to-moderate correlations with strength on the weaker MS limb + CTL (*r* = 0.49–0.58, *p* = 0.001–0.007).

**Discussion:** In people with MS, the single-leg heel raise test may be clinically useful as it identified impaired muscle performance and differentiated muscle performance from a healthy control group and, together with the control group, correlated with functional mobility.

## Introduction

Impaired skeletal muscle performance, as defined by decreased strength and/or endurance, is highly prevalent in people with multiple sclerosis (MS) (1, 2) and contributes to mobility limitations (3). Ankle plantarflexion muscle performance may be especially important for mobility in people with MS as it has been shown to be a key contributor to walking performance (4–8). Furthermore, abnormal gait mechanics are strongly influenced by decreased power and insufficient ankle push-off during late stance, both of which result from impairments in ankle plantarflexion muscle performance (9–11). Therefore, improving ankle plantarflexion muscle performance is a common goal of rehabilitation intervention (12–15). However, clinical measurement of plantarflexion muscle performance can be challenging, which can make it difficult to identify impaired muscle performance and assess the effects of intervention.

The generally accepted standards for strength measurement are electromechanical or fixed dynamometry (16). However, feasibility of these methods is limited in a clinical setting due to cost, availability, and time (16). The most common, clinically feasible methods to assess muscle strength are manual muscle testing and handheld dynamometry (17). However, neither manual muscle testing nor handheld dynamometry is a considered valid measure of ankle plantarflexion strength as examiner strength is typically insufficient to overcome ankle plantarflexion force, even in populations with significant ankle plantarflexion weakness (18, 19). This ceiling effect limits the clinical utility of both manual muscle testing and handheld dynamometry when identifying ankle plantarflexion muscle impairment and assessing change following intervention.

Due to the limitations of manual muscle testing and hand-held dynamometry assessments, weight-bearing assessments of ankle plantarflexion muscle performance are commonly used in the clinic (20–22). One such measure is the heel raise test, which measures the total number of single-leg heel raises that a patient can complete in a continuous bout at a consistent cadence (23, 24). Previous studies have reported that in people with MS the heel raise test was reliable for assessing both the weaker and stronger limbs (2), valid at differentiating between people with MS and a healthy control group (2), and a key contributor to walking performance (7, 8). While ankle plantarflexion strength measured by electromechanical dynamometry has also been shown to correlate with walking performance (3, 4), to our knowledge, the heel raise test and ankle plantarflexion strength testing have not been previously compared within the same study.

Additional insight into the validity of the heel raise test would assist in the clinical assessment of ankle plantarflexion muscle performance and help identify the need for strengthening interventions to improve functional mobility in people with MS. First, it is important to investigate the convergent validity of the heel raise test with a reference standard such as isometric strength measured by electromechanical dynamometry. Second, as it is not clear how the heel raise test compares to electromechanical dynamometry when differentiating between people with MS and healthy controls, it is important to investigate the discriminative validity of both assessments. Third, it is important to examine the association of both the heel raise test and ankle plantarflexion isometric strength with common clinical mobility assessments.

The objectives of this study were therefore to evaluate the validity of the single-leg heel raise test in people with MS and healthy control group participants by determining the (1) convergent validity of the heel raise test with ankle plantarflexion strength assessed by electromechanical dynamometry; (2) discriminative validity of both measures to differentiate between groups; and (3) associations of both measures with walking speed, walking endurance, and stair climbing assessments.

## Materials and Methods

### Participants

This study analyzed cross-sectional data from a group of people with MS (*n* = 21) and an age-and-sex comparable control group (CTL, *n* = 10) (25). Prior to enrollment, all participants signed an informed consent form approved by the local Institutional Review Board. Full eligibility criteria have previously been published (25), but briefly, adults (ages 18–65) with a neurologist-confirmed diagnosis of MS and ability to ambulate 100 m without an assistive device (Expanded Disability Status Scale—EDSS <6) were included. People with MS were excluded if they had more than minimal spasticity (Modified Ashworth Scale ≥ 2 in either lower extremity) as spasticity, especially in the calf muscles, can affect walking and potentially confound the muscle-walking relationship (26). People in the CTL group were matched 1:2 with the MS group (by age and sex) and had no neurologic, muscular, or skeletal disorders. All testing took place in a human performance laboratory on a university medical campus.

Age, height, body mass, and sex were recorded for all participants. The Kurtzke Expanded Disability Status Scale (EDSS) was used to measure disability for the participants with MS (27).

### Outcomes

Ankle plantarflexion muscle performance was assessed *via* the heel raise test (muscle endurance) and electromechanical dynamometry (isometric muscle strength measured *via* peak torque). For the heel raise test, the participants stood with their hands lightly touching a wall at shoulder height and elbows flexed to assist with balance only. While maintaining an extended knee, the participants were asked to perform as many single-leg heel raises as possible with the heel rising at least 5 cm from the floor during each repetition, and a minimum cadence of 40 heel raise repetitions per minute (23). A laser pointer, positioned 5 cm from the ground and positioned just posterior to the medial malleolus with the foot resting on the ground, was used to verify the height of each heel raise. One test was performed on each leg, and the total number of repetitions for each limb was recorded separately and used for data analysis.

Isometric plantarflexion muscle strength was measured using an electromechanical dynamometer (HUMAC Norm, Computer Sports Medicine Inc., MA, USA) with torque data collected at 100 Hz while the participant was in a semi-reclined supine position, with hip flexed to 80 ± 5° and knee flexed to 60 ± 5°, and ankle in 0° dorsiflexion (28, 29). All participants performed five total trials with each trial requiring a 5-s isometric effort. Two initial submaximal familiarization trials were performed followed by three trials at maximal effort. The peak torque value from each trial was recorded, and the average of the three maximal trials was used for data analysis, recorded in torque (Nm), and normalized to body mass (kg).

Functional mobility was measured by the Timed 25-Foot Walk (T25FW), 2-Min Walk test (2MWT), and the Functional Stair Test (FST). The T25FW is a standard measure of gait speed in people with MS where participants walk as quickly and safely as possible for a distance of 25 ft (30). Two trials were performed, averaged together, and reported in m/s for statistical analysis. The 2MWT, a reliable outcome that correlates strongly with the 6-Min Walk test in people with MS, was used to assess gait endurance (31, 32). Participants were asked to cover as much ground as possible in 2 min along a 30-m walkway during a single trial, and the total distance walked was reported in meters. For the FST, participants were timed while ascending one flight of four steps (each step was 23.5 × 76.2 × 15.2 cm) as quickly and safely as possible, using the hand rail(s) as needed (33). Two trials were performed, and the mean, reported to the nearest 1/100th second, was used for data analysis. The T25FW, 6MWT, and FST have all been shown to be highly reliable in people with MS. (30, 31, 33).

All participants performed the outcomes in a standardized order: T25FW was performed first, followed by the single-limb heel raise test, strength testing, FST, and 2MWT. Rests of at least 5 min were mandated between all outcome assessments. Both limbs were assessed for all participants for both the heel raise test and dynamometry. For people with MS, the stronger vs. weaker side was determined based on manual muscle strength testing from the EDSS assessment. The CTL group self-reported dominant limb based on the side they would use to kick a ball, and the average of the CTL group limbs was used for comparisons with the MS group and for associations with mobility measures.

### Data Analysis

Descriptive characteristics were reported for both groups using mean/standard deviation for all characteristics except sex (frequency) and EDSS (median). The sample size for the original trial was calculated to detect differences between groups on kinematic gait variables with a minimum of 10 participants per group (25). This same sample size also provided 83% power (α = 0.05) to detect a difference of at least 13 single-leg heel raise repetitions (SD = 10) between groups, which was the most conservative result from a prior study on the single-leg heel raise test (2). A total of 20 participants with MS were enrolled in an effort to increase power for the correlation analyses.

Convergent validity was assessed using the Pearson's product moment correlation coefficient. For the comparison of the single-leg heel raise test to isometric strength, three comparisons were evaluated: (1) the association within the total sample (*n* = 62 limbs); (2) the association within the MS group (*n* = 42 limbs); and (3) the association within the MS group for limbs that performed <20 single-leg heel raises, as 20 or more single-leg heel raises have been proposed as the threshold for normal muscle performance (20).

Discriminative validity was determined by comparing groups using an unequal variance two-tailed *t*-test. The MS group weak side and strong side were both individually compared to the average of the CTL group values. Mean differences and 95% CIs were reported for both muscle performance assessments. In order to protect against Type I error from the four comparisons, a Bonferroni correction was used resulting in a threshold of α = 0.05/4 = 0.0125.

For associations of both ankle muscle performance assessments with functional mobility assessments, data for the weaker and stronger MS limbs were combined with the average values of both limbs from the CTL group (combined *n* = 31). Pearson's product moment correlations were utilized and considered very strong from 0.90 to 1.00, strong from 0.70 to 0.89, moderate from 0.50 to 0.69, weak from 0.30 to 0.49, and negligible if under 0.30 (34). In order to protect against Type I error from the 12 comparisons, a Bonferroni correction was used resulting in a threshold of α = 0.05/12 = 0.004. All data analyses were performed using SPSS Statistics 26 (IBM, Armonk, NY, USA).

## Results

Age, sex, and BMI were similar between the MS and CTL groups, and the CTL group performed better on all the functional mobility tests (Table 1). The median EDSS score was 3.5, and the median time since diagnosis was 11 years. There were no missing data.

**Table 1:** Demographics and functional mobility descriptors of MS group and CTL group.

**Measure**	**MS (*n* = 21)**	**CTL (*n* = 10)**
Age (y, mean ± SD)	49 ± 12	48 ± 12
Sex (F, %)	16 F (76%)	7 F (70%)
BMI (kg/m^2^, mean ± SD)	24.6 ± 5.3	23.7 ± 4.2
EDSS (median, range)	3.5 (1.5–5.5)	–
Time since diagnosis (y, median, range)	11 (3–35)	–
T25FW (m/s)	1.23 ± 0.24	1.95 ± 0.20
2MWT (m)	138.7 ± 31.0	219.1 ± 24.1
FST (s)	3.44 ± 0.73	1.66 ± 0.22

*MS, multiple sclerosis; CTL, control; EDSS, expanded disability status scale; T25FW, timed 25-foot walk; 2MWT, 2-min walk test; FST, functional stair test*.

**Figure 1:**
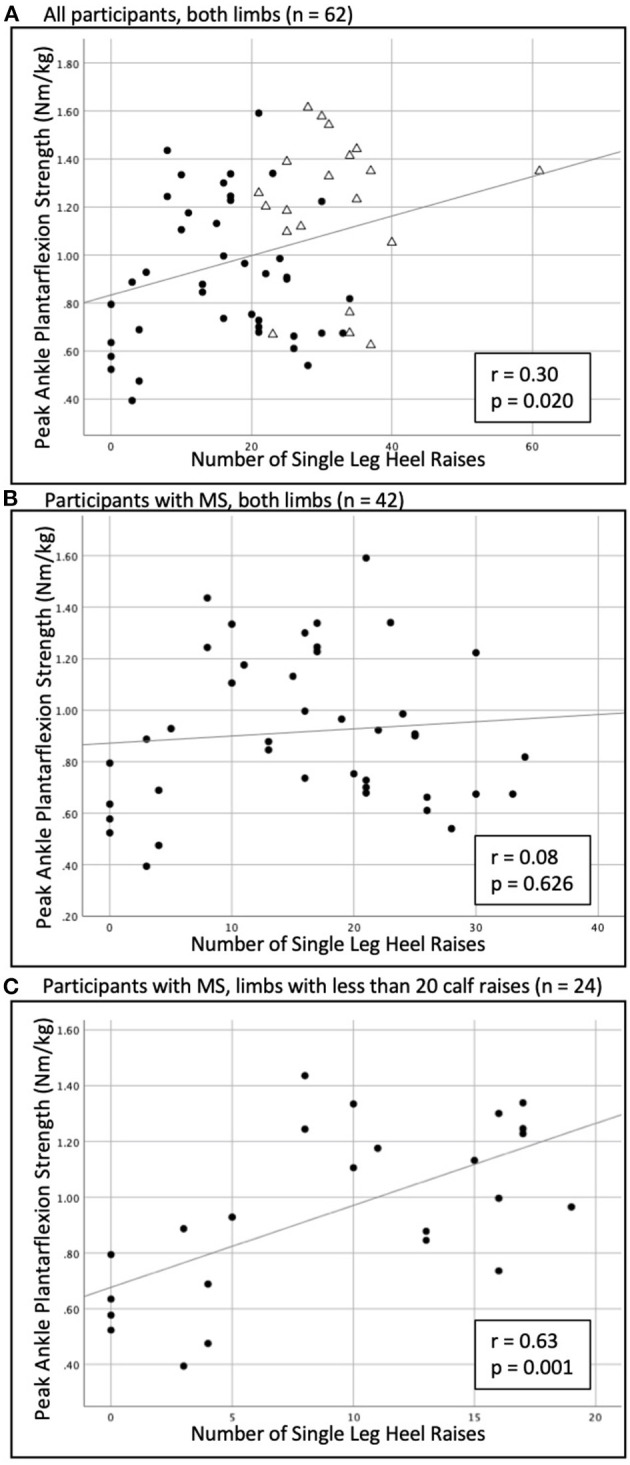
Relationship of the single-leg heel raise test to plantarflexion isometric strength in **(A)** the overall sample (*n* = 62), **(B)** the MS group only (*n* = 42), and **(C)** considering any limb that completed <20 heel raises (*n* = 24). • = participants with MS; △ = healthy control.

Considering all limbs in the overall sample (*n* = 62), the single-leg heel raise test had weak convergent validity with ankle plantarflexion strength (*r* = 0.30, *p* = 0.020, Figure 1). For the MS group only, considering both weaker and stronger limbs (*n* = 42), there was a negligible association between the single-leg heel raise test and ankle plantarflexion strength (*r* = 0.08, *p* = 0.626, Figure 1). In the MS group, considering any limb that completed <20 single-leg heel raises (*n* = 24), there was a moderate association between the single-leg heel raise test and ankle plantarflexion strength (*r* = 0.63, *p* = 0.001, Figure 1).

For discriminative validity (Table 2), there were differences between the weak limb in the MS group and CTL group average for single-leg heel raise repetitions (*p* < 0.001) and for strength (*p* = 0.009). There was also a difference between the strong limb in the MS group and the CTL group average for single-leg heel raise repetitions (*p* = 0.001, Table 2), but not for strength (*p* = 0.081).

**Table 2:** Comparison of muscle performance assessments between the MS group (weak and strong sides) and average CTL group values.

**Measure**	**MS group *N* = 21**	**CTL group average *n* = 10**	**Difference MS vs. CTL (95% CI)**	***p*-value**
Weak side MS Heel Raise to CTL (reps)	12.0 ± 9.4	31.8 ± 7.1	19.8 (13.5 to 26.0)	<0.001^*^
Strong side MS Heel Raise to CTL (reps)	20.7 ± 8.6		11.1 (5.0 to 17.2)	0.001^*^
Weak side MS Strength to CTL (Nm/kg)	0.85 ± 0.26	1.19 ± 0.31	0.34 (0.10 to 0.58)	0.009^*^
Strong side MS Strength to CTL (Nm/ kg)	0.97 ± 0.31		0.22 (-0.03 to 0.47)	0.081

* *Significant based on Bonferroni corrected α = 0.05/4 = 0.0125*.

*MS, multiple sclerosis; CTL, control; reps, repetitions; Nm, Newton-meters; kg, kilograms*.

**Figure 2:**
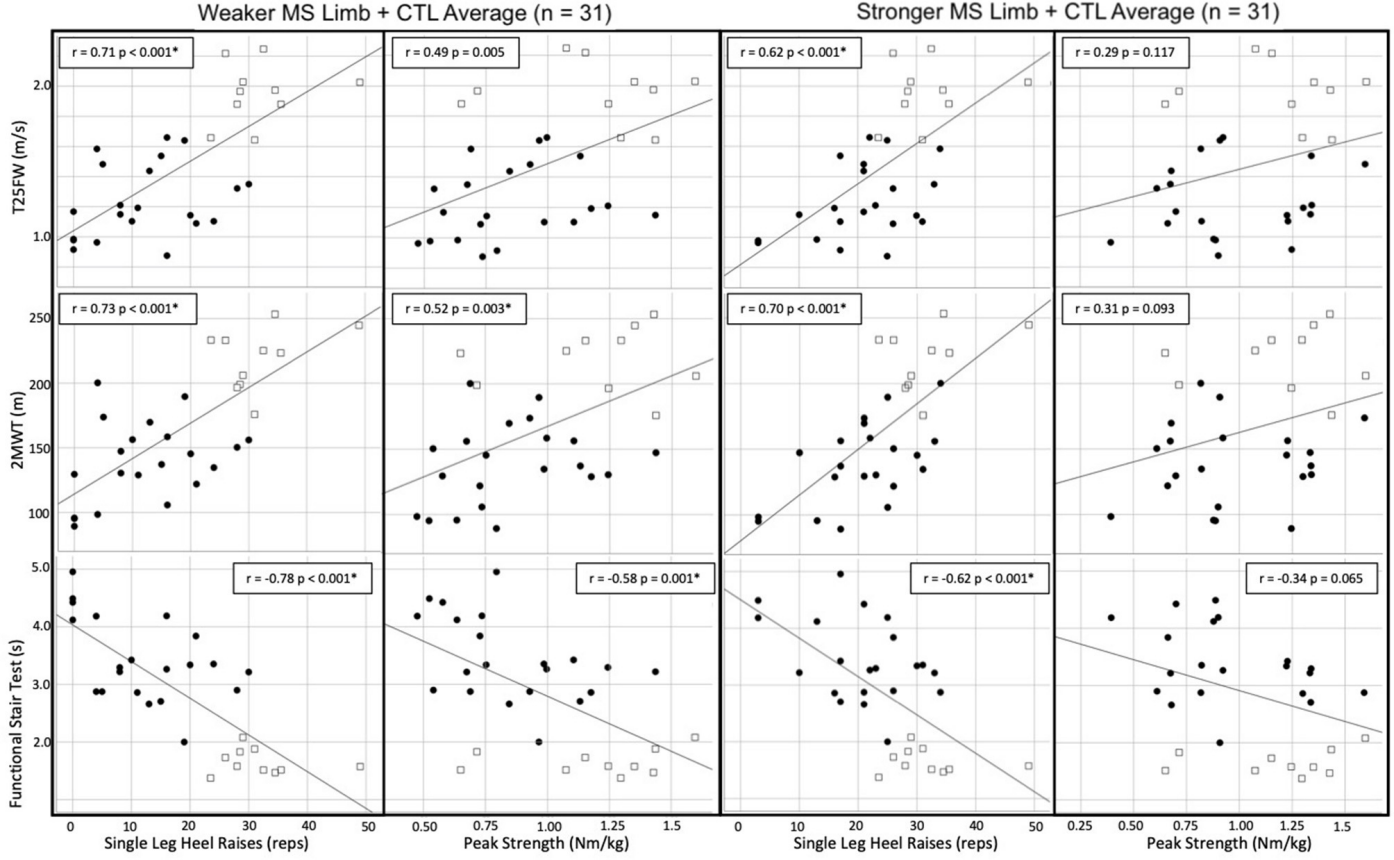
Scatter plots and Pearson values for ankle plantarflexion muscle performance measures to functional mobility. • = participants with MS; □ = healthy control. *Significant based on Bonferroni corrected α = 0.05/12 = 0.004. T25FW, timed 25-foot walk; 2MWT, 2-min walk test; FST, functional stair test.

The associations (Figure 2) of the single-leg heel raise test on the weaker MS limb + CTL (*n* = 31) with functional mobility measures were strong for T25FW (*r* = 0.71, *p* < 0.001), 2MWT (*r* = 0.73, *p* < 0.001), and FST (−0.78, *p* < 0.001). The correlations for the single-leg heel raise test on the stronger MS limb + CTL (*n* = 31) with functional mobility measures were moderate for T25FW (*r* = 0.62, *p* < 0.001) and FST (−0.62, *p* < 0.001), and strong for 2MWT (*r* = 0.70, *p* < 0.001). The associations for ankle plantarflexion strength on the weaker MS limb + CTL (*n* = 31) with functional mobility measures were weak and not significant after adjustment for T25FW (*r* = 0.49, *p* = 0.005), but moderate and significant for 2MWT (*r* = 0.52, *p* = 0.003), and FST (−0.58, *p* = 0.001). Finally, there were no significant correlations for ankle plantarflexion strength on the stronger MS limb + CTL (*n* = 31) with T25FW (*r* = 0.29, *p* = 0.117), 2MWT (*r* = 0.31, *p* = 0.093), or FST (*r* = −0.34, *p* = 0.065).

## Discussion

This investigation determined the validity of the single-leg heel raise test as compared to electromechanical dynamometry, a reference-standard measure of ankle plantarflexion isometric muscle strength. Overall, the single-leg heel raise test did not show strong convergent validity with electromechanical dynamometry. However, the single-leg heel raise test was able to differentiate between people with MS and the CTL group for both weaker and stronger limbs, whereas strength assessment only identified differences between the groups for the weaker MS limb and the CTL average values. Finally, associations of the single-leg heel raise test from the combined groups with functional mobility outcomes were consistently stronger than plantarflexion isometric strength assessment with the same outcomes. The single-leg heel raise test is a simple, clinically feasible muscle performance assessment that has previously been shown to be reliable in people with MS (2). The results from the current study support its validity for clinical evaluation and assessment of ankle plantarflexion muscle performance in people with MS.

The single-leg heel raise test is a measure of muscle endurance, and therefore, it follows that we did not find strong correlations with isometric strength as measured by electromechanical dynamometry. The association of isometric strength to the single-leg heel raise test for the total sample was comparable to a previous study in 43 people with inclusion body myositis, where the heel raise test only explained 13% of variance in maximal strength as tested by fixed dynamometry (19). Furthermore, in the current study, when considering only the limbs of the people with MS, the single-leg heel raise repetitions were not associated with isometric plantarflexion strength. However, when we examined only limbs that performed <20 repetitions (all from the MS group), the single-leg heel raise test was moderately correlated with strength. As the criterion of 20 repetitions has been proposed as normal muscle performance on the single-leg heel raise test (20), this finding suggests that in people with MS who have impaired muscle performance, the single-leg heel raise test may provide some insight into the degree of ankle plantarflexion weakness.

In the current study, the single-leg heel raise test was also able to discriminate between the MS and CTL groups. These findings support a prior study where the single-leg heel raise test was able to differentiate between a group of people with MS (EDSS 1.5 to 5.0) and a healthy comparison group (*n* = 25 per group) (2). Previous studies with larger samples have found isometric plantarflexion strength tested with electromechanical dynamometry to discriminate between people with MS and healthy control participants (4, 10, 14). Therefore, the small sample size of our study might be a reason why we did not detect differences between the groups for the stronger/dominant side comparison. While our findings do not conclude which test is superior, they do suggest that the single-leg heel raise test may be feasible and sufficient in the clinical setting to identify impaired muscle performance in people with MS.

Supporting the clinical utility of the single-leg heel raise test in this study were the moderate-to-strong correlations to functional mobility. Meanwhile, strength as measured by dynamometry had only weak-to-moderate correlations with mobility measures for the weaker MS limb + CTL and weak correlations for the stronger MS limb + CTL. While this study combined the MS and CTL group limbs, prior literature consistently demonstrates that muscle performance on the weaker side correlates more strongly to mobility than the stronger side in people with MS (3). Prior studies in people with MS have also reported moderate associations of both ankle plantarflexion strength (*r* = 0.54, *p* < 0.001) (4) and the single-leg heel raise test (*r* = 0.52 to 0.62, *p* < 0.001) (7, 8) to functional mobility outcomes. We included both the MS and control groups in our correlations, and this is likely why our associations were overall stronger than in prior studies, as the increased variability of the scores and larger sample increased the strength of correlation. Therefore, while our findings should not be directly compared to prior work in MS, they do provide important information about the heel raise test and that it may be at least as good of an indicator of functional mobility as ankle plantarflexion strength measured by electromechanical dynamometry. Given the logistical advantages of the single-leg heel raise test compared to dynamometry, clinicians may thus consider using the single-leg heel raise test to identify people with MS who might benefit from improving ankle plantarflexion muscle performance as a means to improve mobility.

This study had several limitations. One limitation is the floor effect of the single-leg heel raise test. In this study, three participants with MS were unable to complete any single-leg heel raises on the weaker limb, yet all were able to generate force during dynamometry assessment. Future studies are needed to examine a clinically feasible muscle assessment that can assess a wide range of patients with impaired ankle plantarflexion muscle performance. Second, the heel raise test may also be affected by strength in the midfoot, so that it may also explain the low associations with strength assessment. Third, while we decided on a cutoff of 20 heel raises for “normal” strength in order to analyze a specific subset of people with MS, “normal” values for the single-leg heel raise test are not definitively established. However, no single cutoff for the heel raise test is likely to exist, as age, sex, BMI, and even physical activity level can influence the total number of heel raises (20), and a value of 20 seemed appropriate for this study as the minimum value for our control group was 21 repetitions. Fourth, our associations between muscle performance and functional mobility were considerably stronger than what has previously been reported in the literature that only reports values for people with MS, and this is likely because of including the healthy control group. Finally, the small sample size and the fact that all participants with MS could ambulate without assistance further limit the generalizability of the results.

## Conclusion

The single-leg heel raise test had a moderate correlation with plantarflexion strength for people with MS and impaired ankle muscle performance. Additionally, the single-leg heel raise test was able to discriminate between people with MS and a control group. Finally, the single-leg heel-raise test had significant correlations with each functional mobility test, suggesting that the single-leg heel raise test can capture meaningful functional constructs for people with MS. Therefore, while the single-leg heel raise test should not be considered a surrogate of ankle plantarflexion strength, it may have clinical utility in identifying impaired ankle plantarflexion muscle endurance and as an indicator of functional mobility in people with MS.

## Data Availability Statement

The datasets presented in this article are not readily available because of ongoing analysis. Requests to access the datasets should be directed to mark.manago@cuanschutz.edu.

## Ethics Statement

The studies involving human participants were reviewed and approved by Colorado Multiple Institutional Review Board, University of Colorado. The patients/participants provided their written informed consent to participate in this study.

## Author Contributions

All authors listed have made a substantial, direct and intellectual contribution to the work, and approved it for publication.

## Conflict of Interest

The authors declare that the research was conducted in the absence of any commercial or financial relationships that could be construed as a potential conflict of interest.
